# Global distribution and genomic characteristics of linezolid resistance gene-positive *Enterococcus faecium* among humans, animals, and the environment

**DOI:** 10.3389/fmicb.2026.1916332

**Published:** 2026-08-10

**Authors:** Jinxiang Zeng, Ying Wu, Yao Li, Weifeng Lv, Junwei Lin, Qianying Nie, Qi Lin, Xiaoqing Wen, Kun Lin, Lifeng Zhang, Shengzhen Wu, Rong Su

**Affiliations:** 1 The Eighth Clinical Medical College of Guangzhou University of Chinese Medicine, Foshan, Guangdong, China; 2 Department of Laboratory Medicine, Foshan Hospital of Traditional Chinese Medicine, Foshan, Guangdong, China; 3 Foshan Key Laboratory of Chinese Medicine, Foshan Hospital of Traditional Chinese Medicine, Guangzhou University of Chinese Medicine, Foshan, Guangdong, China; 4 Guangdong Provincial Clinical Research Center for Laboratory Medicine, Guangzhou, Guangdong, China

**Keywords:** *Enterococcus faecium*, genomics, linezolid resistance gene, molecular epidemiology, transmission

## Abstract

**Background:**

Over the past decade, considerable attention has been directed toward the plasmid-mediated dissemination of linezolid resistance determinants among *Enterococcus faecium* (*E. faecium*). Despite extensive epidemiological research, a systematic global genomic analysis of linezolid resistance gene-positive *E. faecium* (LRGPEfm) is lacking.

**Methods:**

This study conducted a comprehensive genomic analysis of 832 LRGPEfm genomes, with the aim of investigating their antibiotic resistance genes (ARGs), virulence factor genes (VFGs), population structure, and transmission pathways.

**Results:**

The greatest proportion of LRGPEfm originated from China (32.57%), with Germany following at 11.54%. In relation to host origin, humans represented the dominant category (48.08%), followed by pigs (10.58%). LRGPEfm additionally functions as a repository for ARGs, with a total of 47 ARGs identified. Isolates from China and pigs harbored the highest ARG numbers. Furthermore, 35 VFGs were identified, with isolates from hospital environments and Germany harboring the highest VFG numbers. A total of 211 sequence types were delineated through multilocus sequence typing (MLST) analysis, with ST80 being the most common. Minimal SNP differences detected in LRGPEfm originating from various countries and host sources suggest cross-border and cross-species clonal dissemination. Pan-genome-wide association studies (pan-GWAS) demonstrated that the accessory genome of LRGPEfm varies significantly by geography and host, showing a marked enrichment of genes associated with metabolic enzymes, mobile genetic elements (MGEs), and ARGs. Among the genetic contexts of linezolid resistance genes, IS*1216E* was the predominant carrier.

**Conclusion:**

LRGPEfm poses a substantial public health threat, and sustained, comprehensive surveillance is essential.

## 1 Introduction

Antimicrobial resistance (AMR) has evolved into one of the most pressing challenges confronting contemporary medicine, jeopardizing both public health and food systems on an international scale (Lautan et al., 2025; GBD 2021 Antimicrobial Resistance Collaborators, 2024). Widespread antibiotic misuse has further exacerbated the worldwide AMR crisis (Van Boeckel et al., 2014). Genes that mediate resistance to clinically critical antibiotics, including colistin, carbapenems, and oxazolidinones, merit particular attention (Lu et al., 2025; Wu et al., 2026).

Linezolid resistance is commonly attributed to alterations in ribosomal proteins L3 and L4 (Mendes et al., 2010) and nucleotide changes within domain V of 23S rRNA (Tsiodras et al., 2001). Nevertheless, the rise and propagation of mobile linezolid resistance genes, specifically *optrA*, *poxtA*, and *cfr*, as well as their variants, endanger public health systems on a global scale (Schwarz et al., 2021; Brenciani et al., 2022). The *optrA* gene, first described in *Enterococcus faecalis* from China, encodes an ATP-binding cassette protein belonging to the ABC-F family, mediating resistance to both oxazolidinones and phenicols (Wang et al., 2015). The *poxtA* gene, originally recovered from methicillin-resistant *Staphylococcus aureus* (Antonelli et al., 2018), has subsequently been found in *Ligilactobacillus* and *Enterococcus* (Zhu et al., 2022), and shares 32% homology with *optrA*. The *cfr* gene family, which includes *cfr(B)*, *cfr(C)*, *cfr(D)*, and *cfr(E)* (Deshpande et al., 2015; Tang et al., 2017; Stojković et al., 2019; Pang et al., 2020), encodes rRNA methyltransferases that inhibit protein synthesis; these genes give rise to the PhLOPSA phenotype, characterized by broad-spectrum cross-resistance to pleuromutilins, lincosamides, oxazolidinones, phenicols, and streptogramin A antibiotics (Long et al., 2006).

*Enterococcus faecium* (*E. faecium*) operates as an opportunistic pathogen colonizing the gastrointestinal tract of both humans and animals (Wei et al., 2024). This microorganism has the potential to provoke critical infections in immunocompromised subjects (Fiore et al., 2019). Following the initial detection of vancomycin-resistant *E. faecium* (VREfm), its prevalence has exhibited a progressive rise, which has greatly narrowed the available antibiotic treatment options (Ayobami et al., 2020). This trend highlights the essential function of linezolid in managing infections caused by VREfm (Lu et al., 2025). Recently, however, *E. faecium* has become an important reservoir for linezolid resistance genes (Shen et al., 2024b), and linezolid resistance gene-positive *E. faecium* (LRGPEfm) isolates are widely distributed among various hosts, indicating the possibility of clonal transmission among different hosts (Ocejo et al., 2024; Abraham et al., 2026; Xia et al., 2026). Nevertheless, comprehensive global genomic analysis of LRGPEfm remains insufficient (Beh et al., 2025; Yuan et al., 2026). The present investigation seeks to comprehensively characterize global LRGPEfm isolates by employing advanced analytical approaches, and the findings may provide a conceptual foundation for understanding the distribution and dissemination of linezolid resistance genes across different countries and hosts.

## 2 Materials and methods

### 2.1 Screening linezolid resistance gene-positive *Enterococcus faecium* in the database

As of February 5, 2025, *E. faecium* isolates carrying any of the linezolid resistance genes (*optrA*, *poxtA*, or *cfr*) were downloaded from the public NCBI Pathogen Database. In total, we compiled 832 isolates of LRGPEfm and recorded corresponding metadata, including isolate identifiers, country, host category, and the dates on which the samples were obtained. *De novo* genome assembly was performed using SPAdes v3.13.0 to process raw sequence reads retrieved from the NCBI Short Read Archive for isolates without publicly available assembled genomes (Bankevich et al., 2012). All genomes were subjected to quality control using CheckM v1.1.3,^1^ with inclusion criteria set as genome contamination below 5% and genome completeness above 95%.

### 2.2 Bioinformatics analysis

Multilocus sequence typing (MLST) was performed for all isolates using MLST v2.23.0.^2^ The inferred sequence types (STs) were subsequently employed to generate a minimum spanning tree, which was constructed and visualized by PHYLOViZ v2.0 (Nascimento et al., 2017). The ABRicate^3^ was used with the *Efm-Elts* VirulenceFinder database (Roer et al., 2024) and the ResFinder database to annotate virulence factor genes (VFGs) and antibiotic resistance genes (ARGs), respectively. ISfinder was employed to identify and annotate insertion sequences (ISs) (Siguier et al., 2006). Genome annotation for all isolates was carried out using Prokka v1.14.6 (Seemann, 2014). Roary v3.13 was employed to perform the pan-genome characterization (Page et al., 2015). SNP-site was employed to harvest core genome single-nucleotide polymorphisms (cgSNPs) from a concatenated core gene alignment (Page et al., 2016). Using RAxML-ng, a phylogenetic tree was generated based on cgSNPs via the maximum likelihood approach, applying the GTR + G nucleotide substitution model (Kozlov et al., 2019), and the tree was rendered and annotated using iTOL v6 (Letunic and Bork, 2024). Sequence clusters (SCs) were delineated using Fastbaps v1.0.8 (Tonkin-Hill et al., 2019). Pan-genome-wide association analyses (pan-GWAS) were conducted using pyseer v1.4.1,^4^ with population structure controlled via a linear mixed model (LMM) and multiple-testing correction applied using the Benjamini–Hochberg procedure to reduce the likelihood of false positive results. To investigate the putative clonal linkage of LRGPEfm, pairwise SNP distances were calculated using snp-dists v0.8.2^5^ based on cgSNPs detected by Snippy.^6^ Gephi v0.10.1 was employed to visualize putative clonal linkage networks constructed from pairwise single-nucleotide polymorphism (SNP) distances.^7^ The genetic context of linezolid resistance genes was examined using Flanker v0.1.5 (Matlock et al., 2021). Sequence dereplication was performed with CD-HIT v4.8.1 (Li and Godzik, 2006), and linear comparative gene maps were generated using Lovis4u v0.1.4.1 (Egorov and Atkinson, 2025).

### 2.3 Statistical analysis

The results generated from the preceding analytical steps were extracted and integrated using R software v4.4.2. Linear mixed models were used to evaluate differences in ARG and VFG numbers among different countries or sources, with ST included as a random effect to account for population structure. To profile ARG and VFG constituents among LRGPEfm isolates obtained from different countries or sources, principal coordinate analysis (PCoA) was conducted utilizing Bray–Curtis dissimilarity metrics.

## 3 Results

### 3.1 Overview of the LRGPEfm isolates

**Figure 1:**
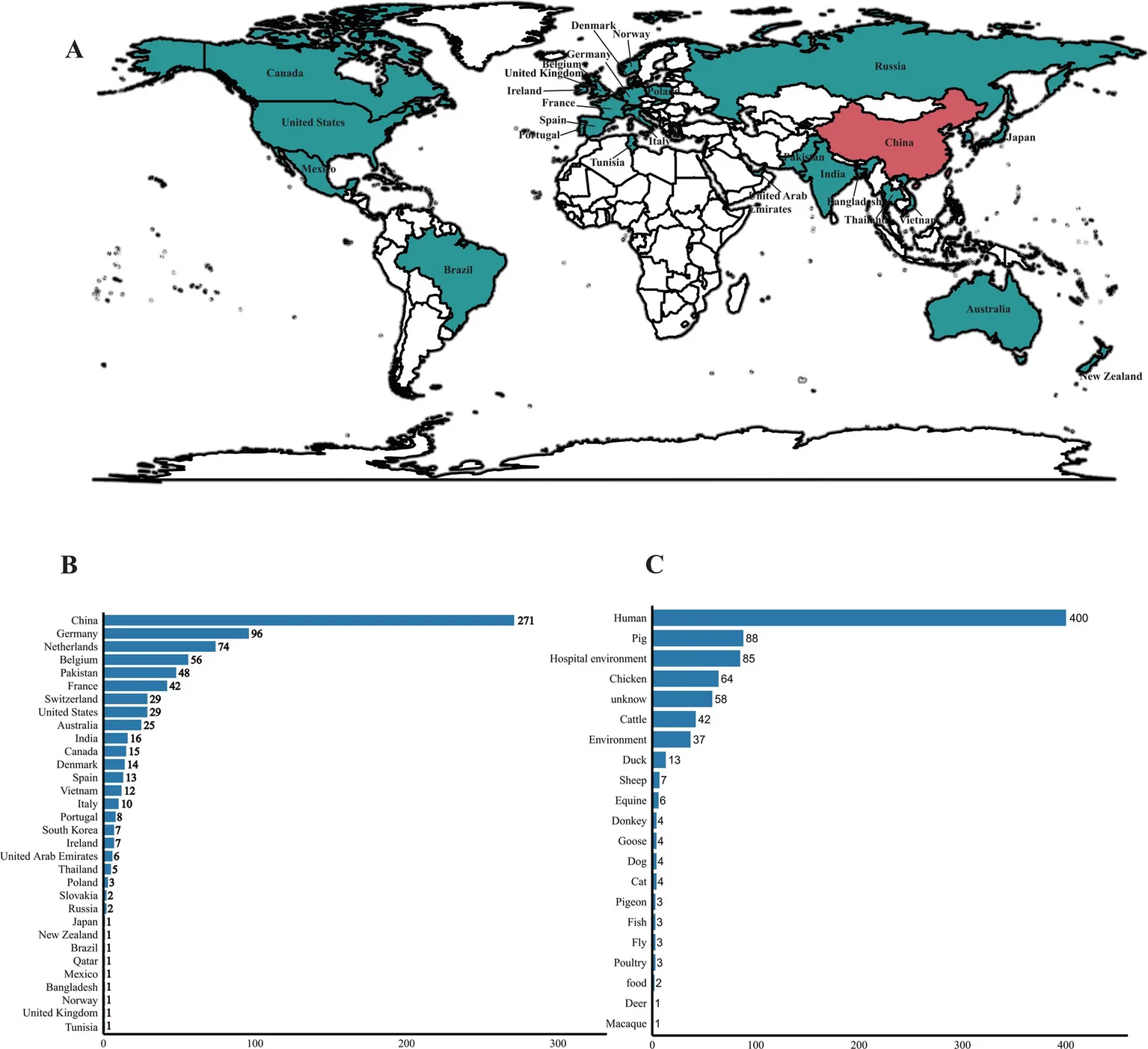
Geographic dissemination of the 832 LRGPEfm isolates worldwide. **(A)** Global geographic dissemination of LRGPEfm. **(B)** Distribution across countries and enumeration of LRGPEfm isolates. **(C)** Distribution across hosts and quantification of LRGPEfm isolates.

A total of 832 nonduplicate linezolid resistance gene-positive *E. faecium* (LRGPEfm) sequences were retrieved from public databases as of February 5, 2025 (Supplementary Table S1). Isolate metadata, including location, host and collection date, was extracted from the NCBI Pathogen Detection database. Geographically, LRGPEfm isolates were most frequently detected in Asia (44.23%, 368/832), followed by Europe (43.03%, 358/832), North America (5.41%, 45/832) and Oceania (3.13%, 26/832). Only one LRGPEfm isolate was reported from each of Africa and South America. The remaining 3.97% (33/832) of the isolates had unspecified regional origins. These isolates were recovered from 32 countries, with the highest prevalence in China (32.57%, 271/832), followed by Germany (11.54%, 96/832) and the Netherlands (8.89%, 74/832). The remaining 47.00% (391/832) were distributed among 29 other countries (Figures 1A,B). These isolates were obtained from 20 distinct sources, classified into three major categories: human-derived, animal-derived and environment-derived. Among these, human-derived isolates constituted the largest proportion (48.08%, 400/832), followed by pig-derived (10.58%, 88/832) and hospital environment-derived (10.22%, 85/832) isolates. Additionally, 6.97% (58/832) of the isolates had unspecified host origins, and the remaining 24.16% (201/832) comprised isolates from other sources, including cattle, poultry, companion animals and various environmental niches (Figure 1C).

### 3.2 Genomic characterization of the LRGPEfm isolates

Among all LRGPEfm isolates, a total of 47 ARGs were detected (Supplementary Table S2). The *optrA* gene was the most frequently detected linezolid resistance gene (53.97%, 449/832), identified in 22 countries and 17 hosts. The *poxtA* gene (41.47%, 345/832) was subsequently identified in 23 countries and 17 hosts. Interestingly, both *optrA* and *poxtA* showed the highest prevalence in China and in human-derived isolates. Additionally, *cfr(D)* (18.75%, 156/832) was identified in 19 countries and 3 hosts, whereas *cfr(B)* (17.91%, 149/832) was exclusively identified in humans from 12 countries. The *cfr(C)* gene was found in only six isolates, and *cfr* in three isolates, being the least prevalent. Notably, co-carriage of multiple linezolid resistance genes was common among the 832 isolates. A total of 181 carried both *optrA* and *poxtA*, 77 carried *optrA* with *cfr(D)*, 51 carried *poxtA* with *cfr(D)*, 3 carried *optrA* with *cfr*, and 36 carried *optrA*, *poxtA*, and *cfr(D)* simultaneously. In addition to linezolid resistance genes, the aminoglycoside resistance gene *aac(6′)-Ii* (99.88%, 831/832) ranked second in prevalence, with the macrolide resistance gene *msr(C)* (92.43%, 769/832) following closely. Additionally, the tetracycline resistance genes *tet(M)* (72.72%, 605/832) and *tet(L)* (60.46%, 503/832), as well as the macrolide-lincosamide-streptogramin B resistance gene *erm(B)* (62.5%, 520/832), were also detected. Notably, the vancomycin resistance genes *vanA* (34.38%, 286/832) and *vanB* (7.45%, 62/832) were also identified.

**Figure 2:**
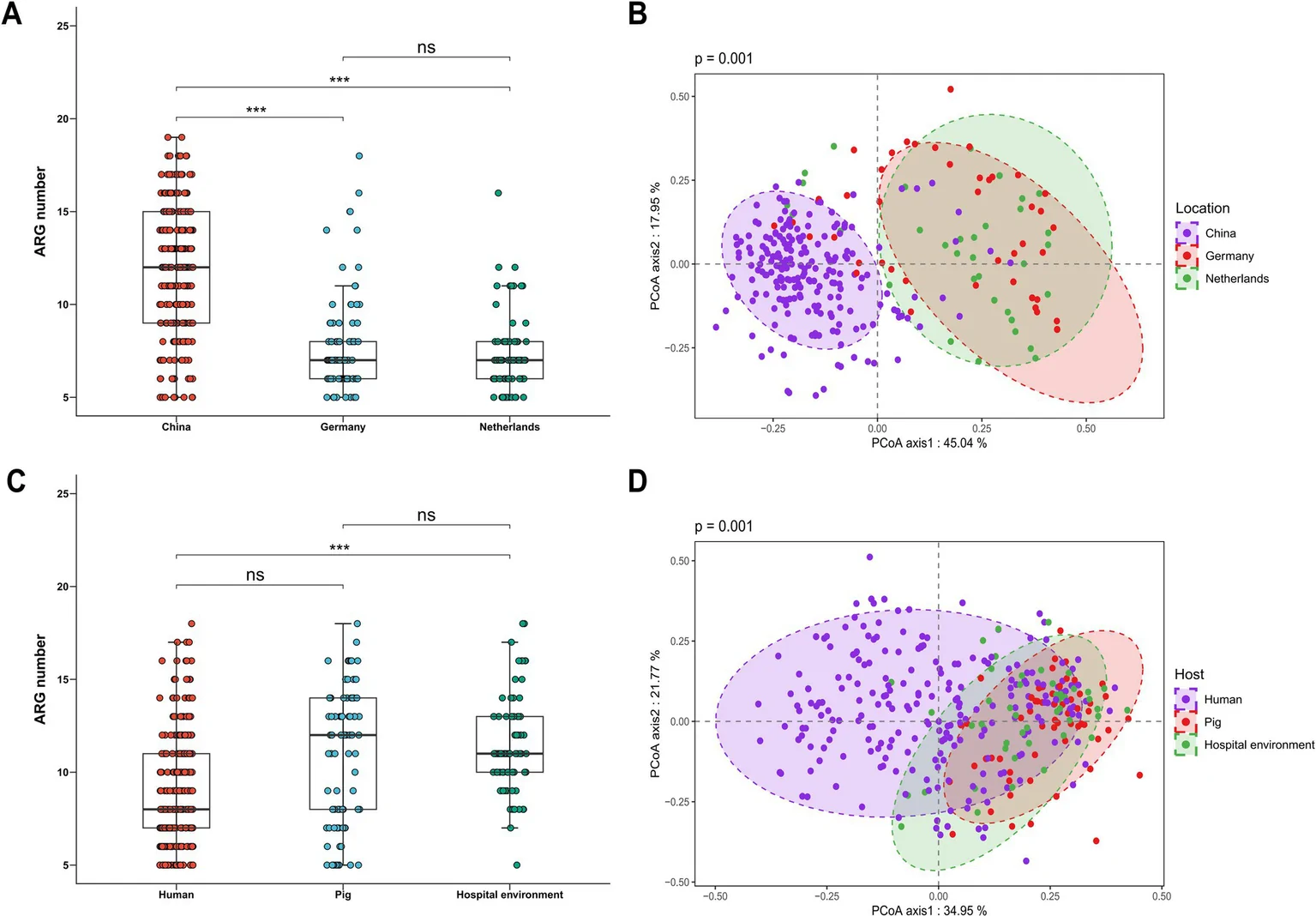
Characterization of ARG distribution among LRGPEfm isolates. **(A)** Differences in ARG numbers across isolates of the three predominant countries. **(B)** PCoA of ARG profiles across the three predominant countries. **(C)** Differences in ARG numbers across isolates of the three predominant hosts. **(D)** PCoA of ARG profiles across the three predominant hosts (Significance: ***p < 0.001; ns denotes no significance).

To characterize variations in the number of ARGs across different countries and hosts, we compared isolates from the three most prevalent countries of origin and the three most prevalent host categories. To address the classic pseudoreplication problem arising from bacterial clonal expansion, we fitted linear mixed-effects models that account for the potential confounding influence of dominant sequence types (STs). The number of ARGs varied considerably depending on the nation of origin. Chinese isolates harbored significantly more ARGs than those from Germany and the Netherlands (*p* < 0.001), whereas no significant difference was observed between German and Dutch isolates (*p* > 0.05) (Figure 2A). Host-specific analysis revealed that pig-derived isolates carried a higher ARG numbers, although the difference from human isolates was not significant (*p* > 0.05), whereas hospital environment-derived isolates carried significantly more ARGs than human-derived isolates (*p* < 0.001), indicating that the hospital environment may serve as an important reservoir of ARGs (Figure 2C). To reveal the distribution patterns of ARGs, a PCoA was performed, as depicted in Figures 2B,D. PCoA revealed that isolates from China displayed a unique distribution pattern of ARG profiles, whereas those from Germany and the Netherlands exhibited highly similar and overlapping patterns, indicating pronounced geographic variation in the ARG profiles of the isolates (Figure 2B). At the host level, the ARG profiles of human-derived isolates showed greater dispersion, whereas those of pig-derived and hospital environment-derived isolates were relatively similar (Figure 2D).

VFGs were highly prevalent across all LRGPEfm isolates, with 20 of the 35 identified VFGs detected in >80% of isolates (Supplementary Table S3). Approximately one-half of isolates (49%) possessed at least 25 VFGs. Adhesion genes, including *fnm* (100.00%, 832/832), *lysM4* (100.00%, 832/832), and *acm* (85.94%, 715/832), were prevalent, along with pilus-associated genes *fms13* (87.98%, 732/832) and *fms17* (76.56%, 637/832). Stress response and metabolic regulators, including the catabolite control protein A gene *ccpA* (100.00%, 832/832) and the general stress response regulators *gls20-glsB* and *gls33-glsB1* (99.64%, 829/832 each), were also frequent. Biofilm and colonization genes, including *sagA* (98.92%, 823/832), *lysM2* (100.00%, 832/832), and *bepA* (99.40%, 827/832), were widely distributed. The biofilm and endocarditis infection protein genes *empA* (83.17%, 692/832), *empB* (88.82%, 739/832), and *empC* (87.86%, 731/832) were similarly prevalent.

**Figure 3:**
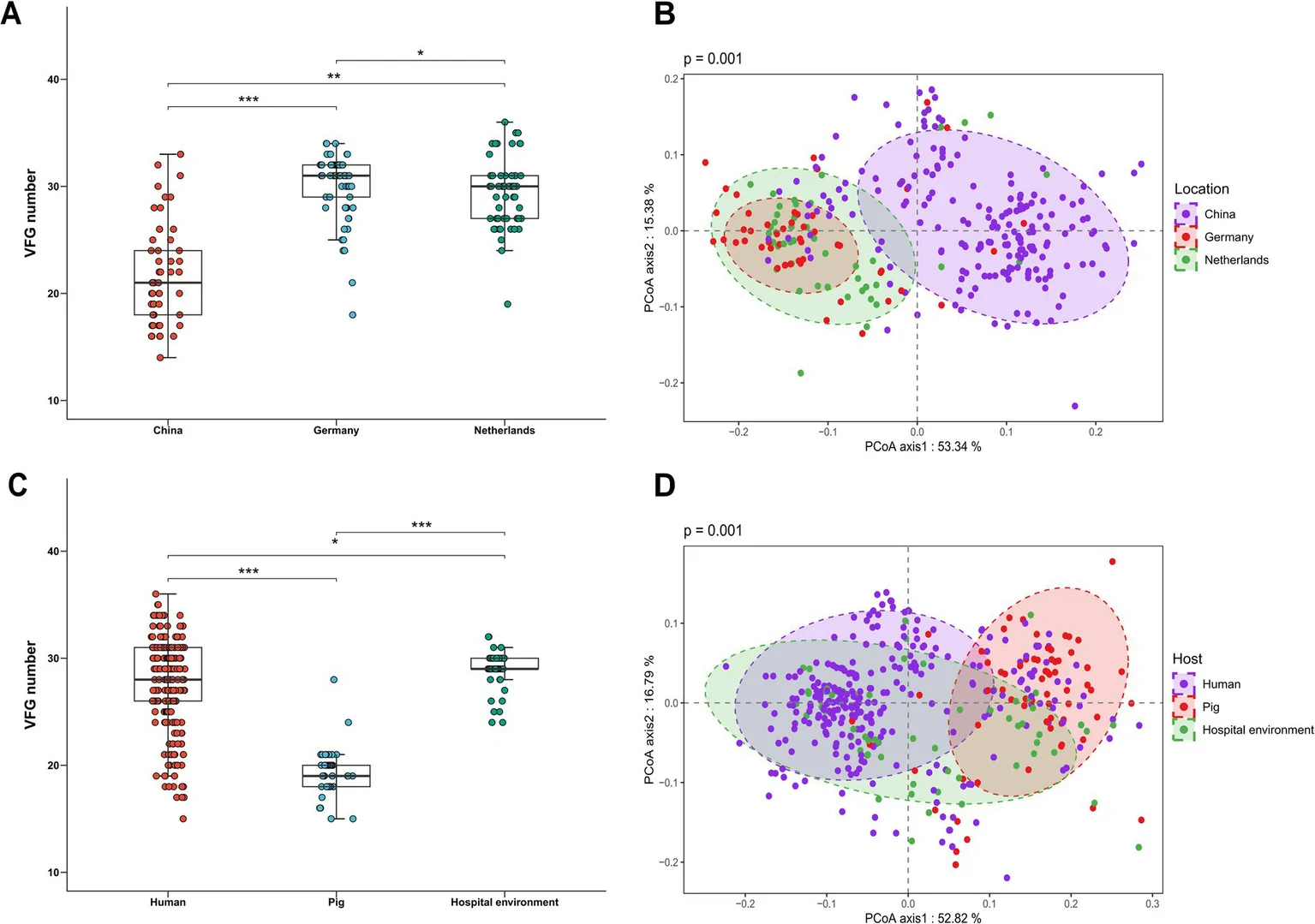
Characterization of VFG distribution among LRGPEfm isolates. **(A)** Differences in VFG numbers across isolates of the three predominant countries. **(B)** PCoA of VFG profiles across the three predominant countries. **(C)** Differences in VFG numbers across isolates of the three predominant hosts. **(D)** PCoA of VFG profiles across the three predominant hosts (Significance: ***p < 0.001, **p < 0.01, *p < 0.05; ns denotes no significance).

Notably, at the country level, isolates from Germany harbored a relatively high number of VFGs. However, isolates from China, which accounted for the largest number of LRGPEfm isolates, carried significantly fewer VFGs (*p* < 0.001) (Figure 3A). Host-specific analysis revealed that hospital environment-derived isolates harbored significantly more VFGs than human-derived isolates (*p* < 0.05) and pig-derived isolates (*p* < 0.001). In addition, human-derived isolates also carried significantly more VFGs than pig-derived isolates (*p* < 0.001) (Figure 3C). To investigate the distribution patterns of VFGs among LRGPEfm from different sources, PCoA was performed. From the country perspective, the distribution pattern of VFGs was similar to that observed for ARGs, with German isolates showing comparable VFG profiles to Dutch isolates, whereas Chinese isolates displayed distinct and wider distribution characteristics (Figure 3B). From the host perspective, hospital environment-derived isolates exhibited a broader VFG distribution, with overlap observed among hospital environment-derived, human-derived, and pig-derived isolates (Figure 3D).

### 3.3 Diversity of STs and phylogenetic analysis of the LRGPEfm isolates

**Figure 4:**
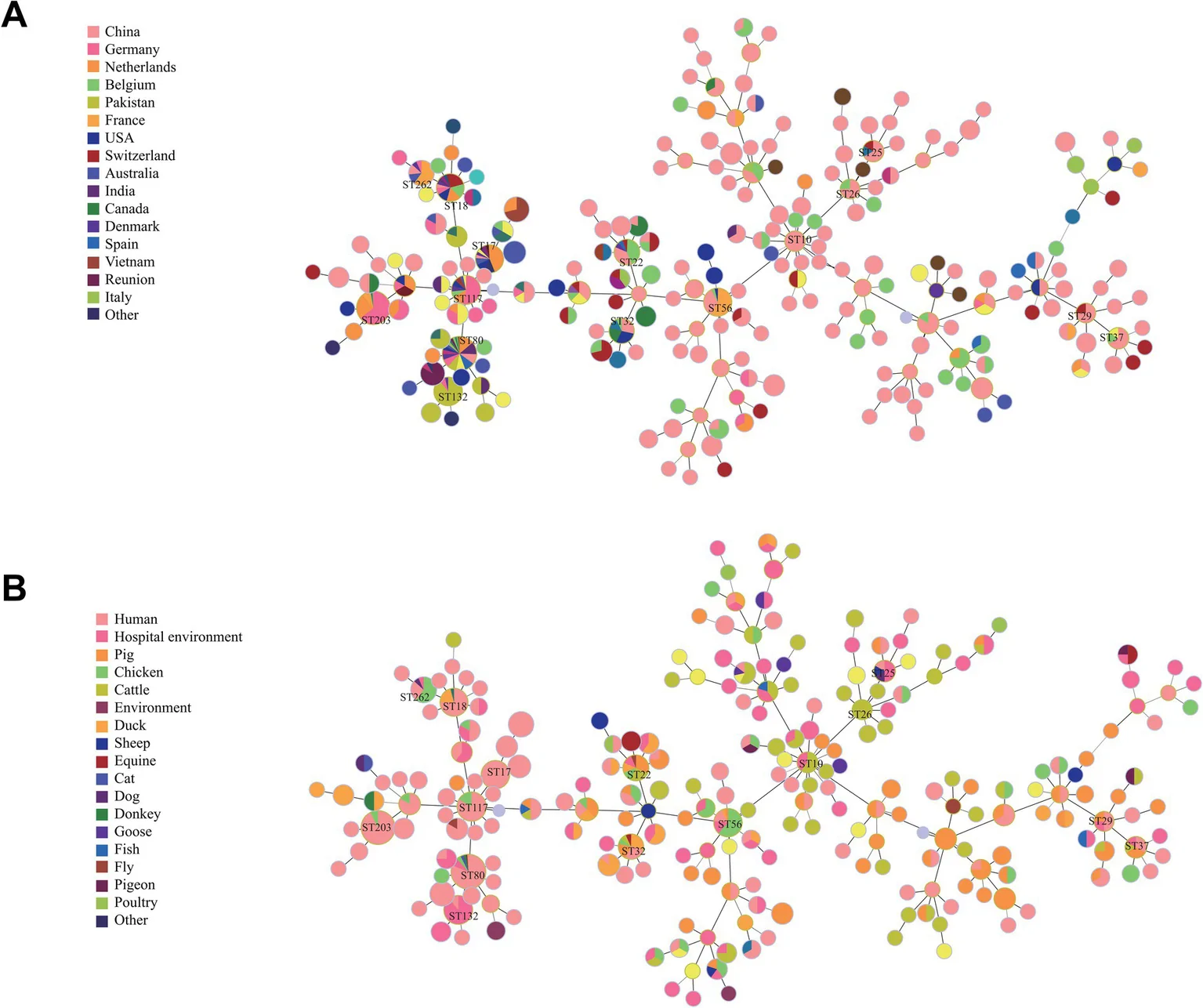
Minimal spanning tree of 832 LRGPEfm isolates. **(A)** Distribution of STs among LRGPEfm originating from various countries. **(B)** Distribution of STs among LRGPEfm originating from different hosts.

Among the 832 LRGPEfm isolates, MLST analysis uncovered remarkable diversity, with isolates classified into 211 known STs, whereas 26 isolates could not be assigned to any known ST (Supplementary Table S1). The most prevalent ST in the LRGPEfm isolates was ST80 (94/832, 11.30%), which was detected in 19 countries and 6 hosts. Other frequently observed STs included ST203 (81/832, 9.74%), which was detected exclusively in human-derived isolates from 5 European countries; and ST117 (30/832, 3.61%), which was exclusively in human-derived isolates from 8 countries. In terms of ST diversity, LRGPEfm isolates recovered from China were characterized by the highest ST richness (59.72%, 126/211), and human-derived isolates exhibited the greatest ST diversity among all host categories (43.60%, 92/211) (Figures 4A,B). These findings indicate that linezolid resistance genes were distributed across a wide range of STs in *E. faecium*.

**Figure 5:**
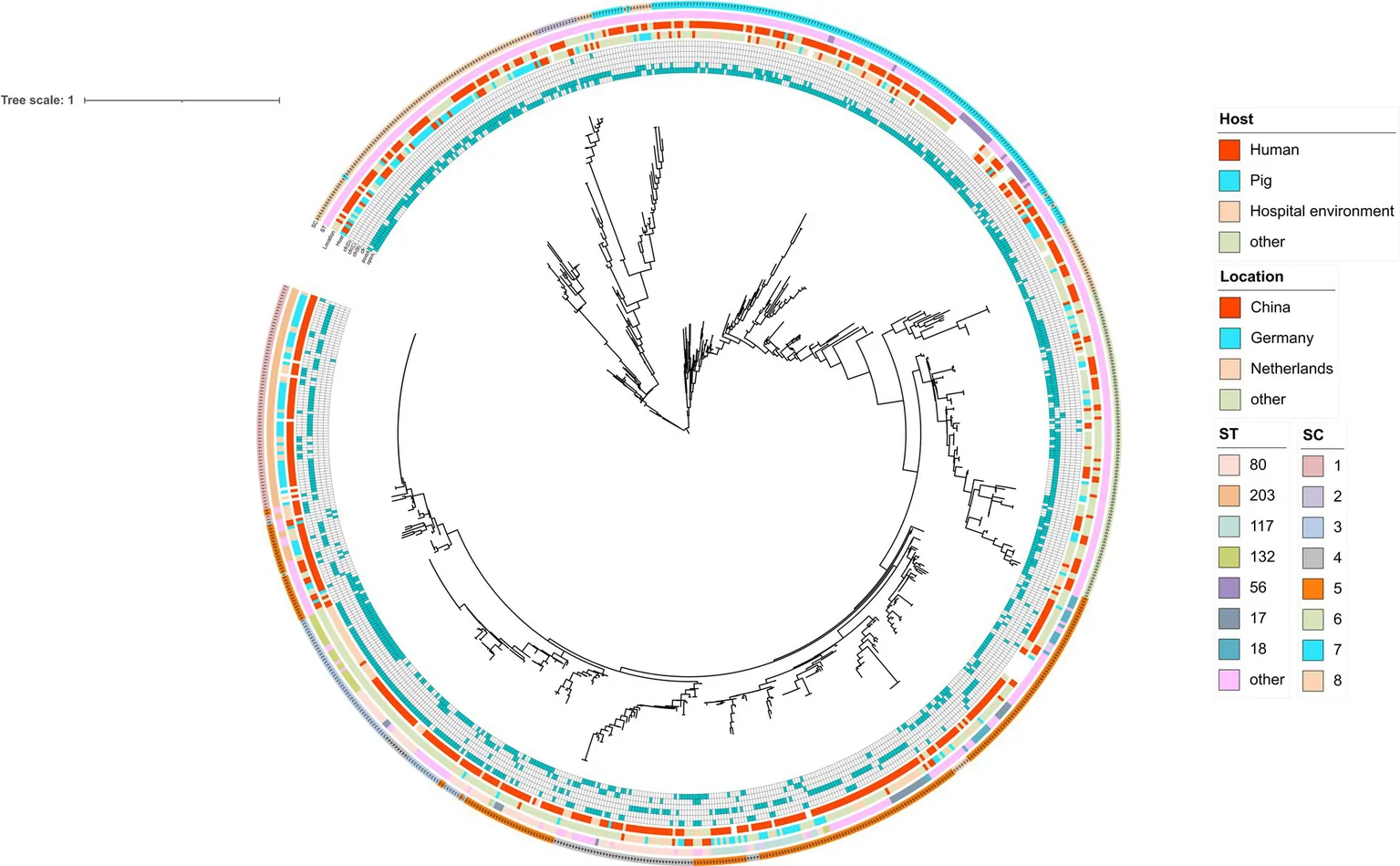
Phylogenetic tree based on cgSNPs of all LRGPEfm isolates. Metadata are displayed on concentric rings according to the legend. From inside to outside: presence of linezolid resistance gene, host of isolation, country of origin, MLST and SC.

To elucidate the phylogenetic relatedness among isolates of varied origins, a pan-genome analysis was conducted across the 832 LRGPEfm genomes, and pairwise SNP distances were subsequently computed for every isolate pair. Among all isolates, a total of 1,060 core genes, 387 soft-core genes, 2,175 accessory genes, and 23,924 unique genes were identified. Additionally, based on hierBAPS analysis, the maximum likelihood (ML) phylogenetic tree of the 832 genomes included eight distinct sequence clusters (SCs) (Figure 5). The most prevalent SC was SC5 (25.98%, 216/832), composed of isolates from 24 countries, followed by SC7 (19.95%, 166/832), which predominantly comprised Chinese isolates, and SC8 (16.23%, 135/832), which contained isolates from 25 countries. Although the LRGPEfm population showed high overall diversity, several SCs exhibited notable geographic or host associations. Specifically, SC1 was predominantly sourced from Germany and the Netherlands and grouped within the same cluster, indicating a strong link to cross-border transmission. Furthermore, SC3 was primarily composed of isolates from hospital environments and human hosts, and some of these isolates displayed close phylogenetic relationships, indicating an underlying risk linked to cross-host clonal spread.

### 3.4 Pan-GWAS analysis of LRGPEfm among different countries and hosts

To explore the distinct distribution of accessory genes across the key countries and hosts, pan-GWAS analyses were carried out using isolates from China, Germany, and the Netherlands and from humans, hospital environments, and pigs. Following the exclusion of hypothetical proteins and the preservation of the top 20 most significant nonredundant hits (Supplementary Table S4), our analysis revealed that key genomic traits were predominantly enriched in metabolic enzymes, mobile genetic elements (MGEs), and ARGs. At the host level, pig-derived isolates exhibited the strongest enrichment of copper resistance-associated genes (*copA* and *copB*), mercury resistance-related genes (*merA* and *merR*) and ARGs (e.g., *aac(6′)-aph(2″)*, *aph(3′)-III* and *poxtA*). The hospital environment-derived isolates were characterized by an enrichment of IS*1252* and ARGs (*tet(M)* and *fexA*), indicating adaptive evolution in response to selective pressures in diverse ecological niches. Human-derived isolates showed fewer strongly enriched accessory genes compared with pig- and hospital-derived isolates. Significant associations were observed mainly for genes involved in cellular metabolism and surface-related functions, including *rfbC*, *gerKI*, *cysE*, and *yvyI*. At the country level, Chinese isolates were enriched for multiple resistance-associated genes, including the florfenicol resistance gene *fexA*, the bacitracin resistance cluster (*bcrR*, *bcrB*, *bcrA*), and MGEs such as IS*1476* and IS*Enfa1*, as well as metabolic genes (galT, uppP, ywrO) and PTS transporter components. German isolates were enriched with *cfr(B)* genes, MGEs (IS*1476* and IS*Efa12*) and the plasmid copy number regulator copG, whereas Dutch isolates were strongly associated with glycopeptide resistance determinants, including the *vanB* cluster, together with *cfr(D)*, *erm(B)*, and IS*256*-associated elements, highlighting region-specific resistance mechanisms.

### 3.5 Putative clonal linkage of LRGPEfm among different countries and hosts

**Figure 6:**
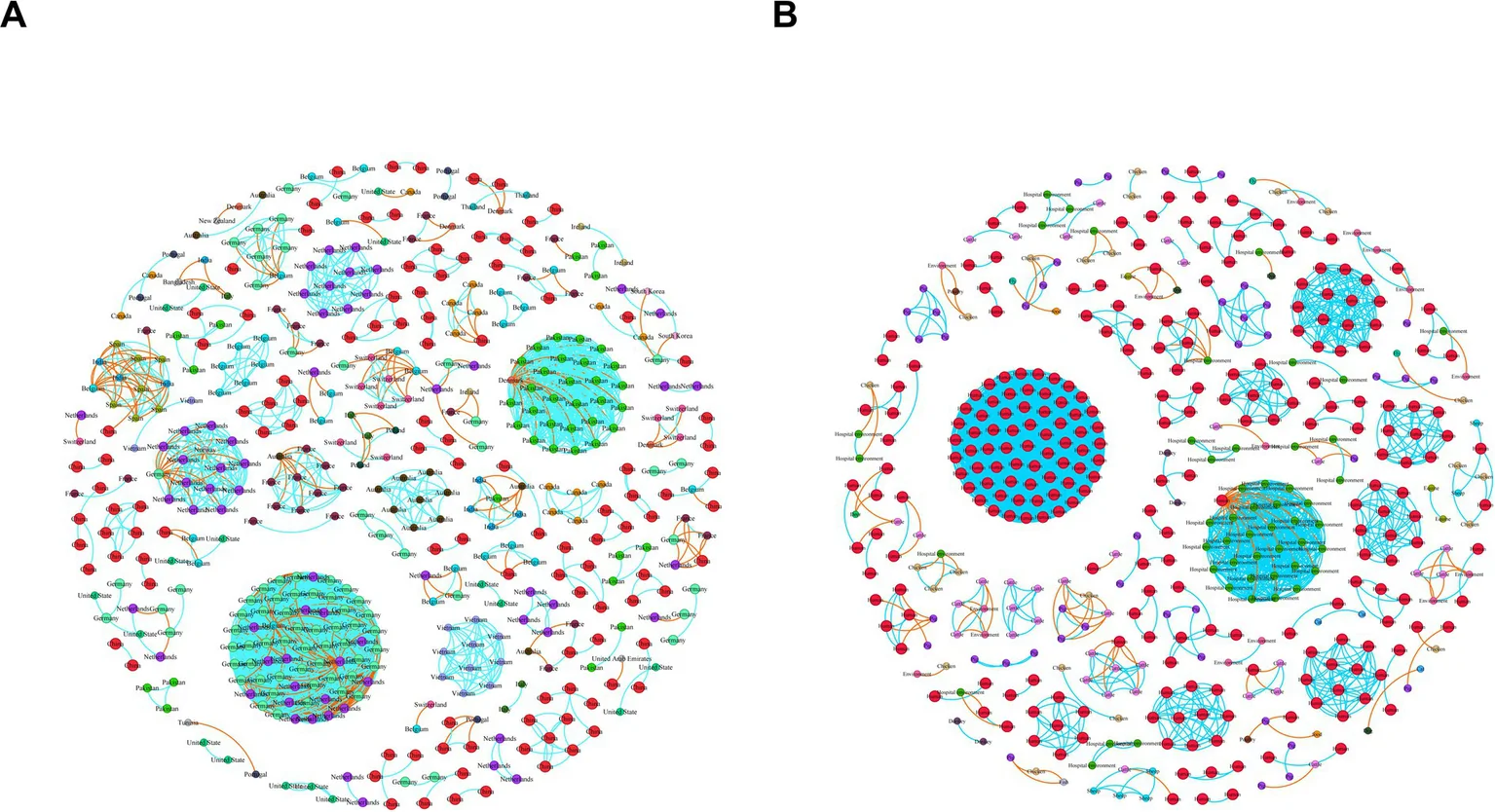
Putative clonal linkage networks were generated among isolates exhibiting ≤25 SNPs. **(A)** Putative clonal linkage network at the country level. **(B)** Putative clonal linkage network at the host level. Node sizes are proportional to the number of isolates involved in collinearity analysis. The blue line represents linkage between the same country or host, while the orange line indicates linkage between different countries or hosts.

To better characterize the close genetic relationships and putative clonal linkages of these LRGPEfm isolates across diverse hosts and locations, a cgSNPs difference matrix was constructed (Supplementary Table S5). Following the criterion established in a preceding investigation (Gorrie et al., 2021), we adopted a pairwise SNP cutoff of ≤25 as a dependable threshold for identifying putative epidemiological linkages in *E. faecium*. A network depicting patterns of clonal relatedness was constructed using Gephi v0.10.1, with nodes weighted by the number of isolates and displaying the major features involved (Figures 6A,B).

At the country level, there are a total of 3,202 putative clonal linkages among 28 countries (Supplementary Table S6). These pairwise linkages were further grouped into two principal categories: (1) within-country clonal linkages, and (2) cross-country clonal linkages. There were 2,131 within-country clonal linkages (54.06% within Germany, 18.25% Pakistan, 12.81% Netherlands and 4.81% China), accounting for 66.55% of the total linkages. Among 1,071 cross-country country clonal relatedness, the largest share (78.24%) was associated with Germany and the Netherlands, representing the predominant pattern. These findings suggest that Germany and the Netherlands share a high degree of cross-border clonal relatedness for LRGPEfm, although the directionality and routes of dissemination cannot be determined from these cross-sectional genomic data.

At the host level, putative clonal linkages amounted to 3,186 (Supplementary Table S7). There were 3,068 within-host linkages (83.74% within humans, 12.91% hospital environments and 1.48% pigs), accounting for 96.30% of the total linkages. Cross-host linkages accounted for 118 events. Among the 64 human-to-other host linkages, 54.68% of them were linked to hospital environments, 23.44% to cattle, and 10.94% to pigs. These findings suggest that closely related LRGPEfm isolates were distributed across multiple hosts and environments, highlighting potential epidemiological connections that require further investigation.

### 3.6 The genetic context of linezolid resistance genes

Contigs carrying linezolid resistance genes were identified and extracted using a custom script. To investigate the genetic context of these genes, we analyzed 5 kbp upstream and downstream regions using Flanker v0.1.5. After redundancy removal using CD-HIT, 19, 38, and 49 distinct flanking environments were identified for *optrA*, *poxtA*, and *cfr*, respectively (Supplementary Figures S1–S3).

A wide variety of *optrA*-carrying genetic platforms were observed, exhibiting considerable diversity in MGEs (Supplementary Figure S1). In this study, IS*Efa15* (*n* = 23) was most commonly recovered from the genetic environment bordering the *optrA* gene, followed by IS*1216E* (*n* = 17). Additionally, other insertion sequences, including IS*1542* (*n* = 3), IS*Efa11* (*n* = 1), IS*Enfa1* (*n* = 1), IS*Sau9* (*n* = 1), and IS*1251* (*n* = 1), were identified in single or multiple copies flanking the *optrA* core region. Transposons such as intact Tn*554* and Tn*5*58, or their remnants of varying lengths, were also identified near the *optrA* gene. The co-occurrence of additional ARGs on these *optrA*-harboring contigs was analyzed, with *erm(A)* (*n* = 266) and *fexA* (*n* = 135) being most commonly detected, thereby facilitating the coselection of *optrA*.

In contrast, the genetic surroundings of the *poxtA* gene were relatively conserved (Supplementary Figure S2). IS*1216E* (*n* = 64) was typically detected adjacent to these contigs, arranged in identical or opposite orientations. Other insertion sequences, including IS*1252* (*n* = 30), IS*1216V* (*n* = 28), and IS*Sag1*2 (*n* = 1), were also present around the *poxtA* core region. Moreover, complete Tn*552* transposons or their derivatives of different sizes were identified in the vicinity of the *poxtA* gene. The co-occurrence of additional ARGs on these *poxtA*-harboring contigs was examined, with *fexB* (*n* = 39) being most commonly detected. With respect to the *cfr* gene, IS*1216E* (*n* = 13) and IS*Efa13* (*n* = 13) were most frequently identified upstream and/or downstream (Supplementary Figure S3). Other flanking insertion sequences included IS*Ssu5* (*n* = 6), IS*1251* (*n* = 2), IS*Enfa1* (*n* = 2), and IS*Sau9* (*n* = 1). Transposons such as Tn*916* or their partial sequences were also detected near the *cfr* gene.

Overall, although the genomic environments surrounding linezolid resistance genes exhibited diversity, they were shared across diverse reservoirs at the source level, indicating horizontal gene transfer and underlying connections among these reservoirs.

## 4 Discussion

Compared with previous investigations, the LRGPEfm genomes collected in our study are more comprehensive, incorporating virtually all available high-quality genomic sequences from various sources. In this study, the global distribution of LRGPEfm across countries and hosts was thoroughly evaluated, and their genomic features were systematically examined. Our findings provide new perspectives regarding epidemiological patterns alongside dissemination profiles of linezolid resistance genes within *E. faecium* recovered from various countries and hosts.

This study found that LRGPEfm was mainly derived from Asia (44.23%) and Europe (43.03%), which aligns with earlier investigations (Dadashi et al., 2021). LRGPEfm was widely distributed across 32 countries, with China contributing the greatest number of positive isolates (32.57%), whereas approximately half of the countries contributed ten or fewer LRGPEfm isolates, which may be linked to disparities in the level of attention given to LRGPEfm. A greater volume of data available from a country allows for a more accurate representation of the true epidemiological landscape.

LRGPEfm isolates were predominantly obtained from humans (48.08%) and pigs (10.58%), underscoring the importance of *E. faecium* both as an opportunistic pathogen under routine surveillance and as a foodborne bacterium. Pig-derived isolates have been identified as an important reservoir of linezolid resistance genes in multiple studies (Wang et al., 2023; Shen et al., 2024b). Notably, although linezolid is prohibited for use in livestock and poultry production (Timmermans et al., 2021), the persistence of LRGPEfm in pig-derived isolates may be attributable to coselection driven by the use of other antibiotics (Zhao et al., 2016; Driesen et al., 2024; Pereira et al., 2025).

The intensifying challenge of LRGPEfm resistance, driven by the innate antibiotic resistance and horizontal transfer of ARGs in *E. faecium*, necessitates the adoption of several innovative therapeutic strategies for managing LRGPEfm infections. The predominant resistance genotypes indicated that most LRGPEfm isolates may exhibit varying degrees of resistance to macrolides, glycopeptides, and tetracyclines. Relevant ARGs, such as *erm(B)* (62.50%), *vanA* (34.38%), *tet(M)* (72.72%), and *tet(L)* (60.46%), showed high detection rates. Of particular concern, the co-carriage of *vanA* and linezolid resistance genes poses a significant threat, as linezolid is a last-resort antibiotic for the treatment of VREfm infections (Miller et al., 2020; Lu et al., 2025). This coexistence may severely compromise therapeutic efficacy and represents a potential public health risk.

Differences in the number of ARGs across geographic regions indicate selection pressure arising from variations in antimicrobial exposure (Ingle et al., 2018). A striking finding was that compared with isolates from other countries, LRGPEfm isolates from China harbored a greater number of ARGs. Moreover, pig-derived LRGPEfm isolates carried the highest number of ARGs among the three sources, although this difference was not statistically significant. This numerical trend, however, may be partly attributable to the intensive antimicrobial selection pressure in livestock production, suggesting that pig-derived LRGPEfm may constitute a potential public health concern (Shen et al., 2024b). Although human-derived LRGPEfm generally carried fewer ARGs, they displayed the most extensive diversity, which is strongly connected to the application of clinical antimicrobial agents. The high burden of ARGs in pig-derived LRGPEfm is consistent with previously published literature (Zhao et al., 2025).

Our analysis revealed that isolates from Germany carried a greater number of VFGs, which was opposite to the pattern observed for resistance genes, suggesting a possible inverse relationship between ARG and VFG diversity that warrants further investigation. Notably, Chinese isolates exhibited a distinct distribution of ARGs and VFGs and formed an independent cluster, the underlying reasons for which require further investigation.

The LRGPEfm isolates analyzed in this study exhibited substantial ST diversity, among which ST80 was the most prevalent and was widely distributed across four continents and 19 countries. In the phylogenetic tree, these isolates mostly clustered with human-derived LRGPEfm, indicating close genetic relatedness, with some isolates differing by fewer than 25 SNPs. Previous studies have shown that ST80 is a common ST in *E. faecium* and has been reported in multiple countries (Pinholt et al., 2019; Egan et al., 2022; Shen et al., 2024a). In contrast to ST80, other STs were absent from multiple continents, suggesting that they may be confined to specific regions and have not disseminated globally. For example, ST203 was the second most prevalent ST and was isolated exclusively in Europe, where it was widely found in Germany and the Netherlands. The majority of ST117 isolates were derived from Europe, with only two identified in the United States, which may be related to independent outbreaks in different countries (Liese et al., 2019; Sabat et al., 2025). Of particular note, China exhibited the highest ST variability among all surveyed countries, suggesting that enhanced surveillance and genomic monitoring of LRGPEfm in this region are warranted.

Pan-GWAS-based analysis of accessory gene variation in isolates collected from diverse origins can uncover the distinctive genetic features harbored by isolates from specific ecological niches during adaptive evolution. In this study, for the pan-GWAS analysis of LRGPEfm, variables such as country of origin and host type were utilized as individual traits. MGEs are pivotal in the transmission of ARGs (Stokes and Gillings, 2011; Partridge et al., 2018). IS*1251* was identified as significantly enriched in MGEs in hospital environment-derived LRGPEfm. The enrichment of these MGEs may partly explain the significantly greater number of ARGs in hospital environment-derived isolates compared with human-derived isolates. Pig-derived isolates were characterized by the coexistence of antimicrobial resistance genes and metal resistance genes. The enrichment of *copA*/*copB* and *merA*/*merR* indicates potential co-selection driven by exposure to heavy metals in agricultural settings. Human-derived isolates showed fewer strongly enriched accessory genes, potentially reflecting lower and more intermittent antimicrobial exposure compared with livestock and hospital settings. Chinese isolates showed the strongest signals for *fexA* and the *bcr* bacitracin resistance cluster, consistent with their quantitatively highest ARG burden. The co-enrichment of IS*1476*, IS*Enfa1*, and metabolic genes in Chinese isolates suggests that MGE-mediated horizontal gene transfer, combined with enhanced metabolic capacity, may offset the fitness cost of maintaining a large plasmid-borne resistome. Compared with LRGPEfm from China, LRGPEfm from Germany possessed IS*1476* and IS*Efa12* as distinct MGEs, whereas Dutch LRGPEfm carried significantly enriched genes *vanB*, *cfr(D)* and IS*256*, which might, respectively, facilitate the horizontal transfer of certain ARGs.

Core genome SNP comparisons revealed small SNP differences among isolates from varied countries and hosts, indicative of close phylogenetic relatedness and putative clonal linkage. Further collinearity analysis of isolates with SNP distances ≤25 indicated that the severity of clonal transmission could have been underestimated in prior studies. LRGPEfm isolates derived from 28 nations demonstrated considerable genomic similarity, indicating potential for clonal dissemination across countries and sources, including livestock, other animals, and hospital environments. We hypothesize that the cross-host and cross-regional dissemination of LRGPEfm is potentially driven by factors such as human-associated movement, livestock transportation, and commercial exchanges. The use of other antibiotics may also exacerbate its dissemination (Zhao et al., 2016; Schwarz et al., 2021; Driesen et al., 2024; Pereira et al., 2025). Of particular note, 78.24% of cross-country putative clonal linkages involved Germany and the Netherlands, suggesting a highly connected transmission network between these two countries. This dominance may be attributed to several factors, including geographic proximity and cross-border patient transfers (Schwarz et al., 2021). Notably, among the cross-host putative clonal linkages, hospital environments were identified as the most frequent recipient of human-derived LRGPEfm, followed by cattle (23.44%) and pigs (10.94%). Collectively, these results reveal that hospital environments serve as a pivotal hub for the dissemination of LRGPEfm, underscoring the intricate nature and significant threat of hospital-acquired clonal transmission (Zhao et al., 2016). Moreover, cattle-derived and pig-derived isolates have been confirmed to be important reservoirs of linezolid resistance genes (Ocejo et al., 2024; Abraham et al., 2026; Xia et al., 2026), and LRGPEfm has also been isolated from market-derived meat and food-producing animals, suggesting that these isolates may disseminate along the food production chain. These factors likely contribute to the widespread dissemination of LRGPEfm across countries. To clarify the global transmission routes of LRGPEfm, continuous monitoring of these factors is essential for identifying the major pathways of dissemination.

MGEs, including integrons, transposons, and ISs, facilitate the horizontal dissemination of ARGs among bacteria, serving as a core mechanism that drives the spread of these loci throughout varied ecosystems and distinct microbial species (Partridge et al., 2018). Numerous MGEs were found in the genomic surroundings of linezolid resistance genes, likely facilitating the horizontal mobilization of these resistance loci. Among these elements, IS*1216E* was particularly notable because it was detected most frequently and has been shown to facilitate the transfer of overlapping clusters harboring the *optrA*, *poxtA*, and *cfr* genes in the form of IS*1216E*-based mobile units (Dejoies et al., 2021; Schwarz et al., 2021; Brenciani et al., 2022). Moreover, these linezolid resistance loci frequently exhibited co-selection with multiple other ARGs. Specifically, the *optrA* gene was found in proximity to several resistance determinants, including *fexA* and *erm(A)*, while the *poxtA* gene was located adjacent to *fexB*. This co-localization suggests that LRGPEfm harboring the *optrA* gene may concurrently exhibit resistance to phenicols (*fexA*-mediated), as well as streptogramins, lincosamides, and macrolides (*erm(A)*-mediated), which underscores the need for heightened vigilance. This may contribute to the sustained maintenance of linezolid resistance genes in *E. faecium*, highlighting a critical need for close surveillance.

Our investigation is subject to a number of limitations. First, because the genomes were retrospectively retrieved from public database, the dataset is affected by substantial geographic and host imbalance. Human-derived isolates predominated, and China contributed the largest national share, whereas Africa, South America, and Oceania were sparsely represented; this overrepresentation of certain regions and hosts may introduce selection bias and compromises the representativeness of the inferred population structure of LRGPEfm. Second, the presence of linezolid resistance genes does not necessarily confer resistance to linezolid; thus, relying solely on genotypic detection may overestimate the severity of linezolid resistance in *E. faecium*. Consequently, more research must be conducted to clarify the mechanistic pathways conferring linezolid resistance. Phenotypic testing was not performed on isolates carrying linezolid resistance genes, preventing exploration of the correlation of genetic determinants with actual resistance profiles. Finally, owing to the lack of long-read sequencing technology, it was difficult to accurately determine the precise locations of ARGs. Future studies integrating third-generation full-length sequencing with thorough plasmid structural characterization will be important for further developing these preliminary insights.

## 5 Conclusion

In this study, a worldwide genomic survey of LRGPEfm retrieved from the NCBI database was conducted, with a focus on genomic data, to support the surveillance and control of linezolid resistance gene dissemination and its public health implications. LRGPEfm isolates originating from China and pig sources accounted for the greatest number of LRGPEfm isolates and harbored distinct arrays of ARGs. The isolates exhibited substantial diversity in STs, with ST80 being the predominant ST. Pan-GWAS analysis indicated that the differentially enriched genes in LRGPEfm were primarily linked to carbohydrate metabolism genes, MGEs, and ARGs. Substantial evidence points to the clonal dissemination of LRGPEfm across diverse geographical regions and different hosts. Analysis of the genetic environment surrounding linezolid resistance genes revealed that various IS elements in the flanking regions may facilitate their dissemination, with the IS*1216* element being particularly prominent. This work demonstrates putative clonal relatedness and epidemiological linkages across different countries and hosts; however, more comprehensive analyses are required to thoroughly elucidate the cycling of linezolid resistance genes within ecosystems. Certain constraints, particularly concerning sample representativeness and source imbalance, are noted in this work. In conclusion, linezolid resistance genes are widely distributed across diverse sources in numerous countries, presenting a substantial hazard, which underscores the need for ongoing monitoring and rigorous containment approaches to protect public health.

## Data Availability

The original contributions presented in the study are included in the article/Supplementary material, further inquiries can be directed to the corresponding author.

## Footnotes

1
https://github.com/Ecogenomics/CheckM

2
https://github.com/tseemann/mlst

3
https://github.com/tseemann/abricate

4
https://github.com/mgalardini/pyseer

5
https://github.com/tseemann/snp-dists

6
https://github.com/tseemann/snippy

7
https://gephi.org/
