# Supplementary material for: Global distribution and genomic characteristics of linezolid resistance gene-positive Enterococcus faecium among humans, animals, and the environment
Source: Front Microbiol. 2026 Aug 10;17:1916332. doi: 10.3389/fmicb.2026.1916332 (PMC13501226; doi:10.3389/fmicb.2026.1916332)
Supplement: Supplementary file 1 [file Image_1.PDF]

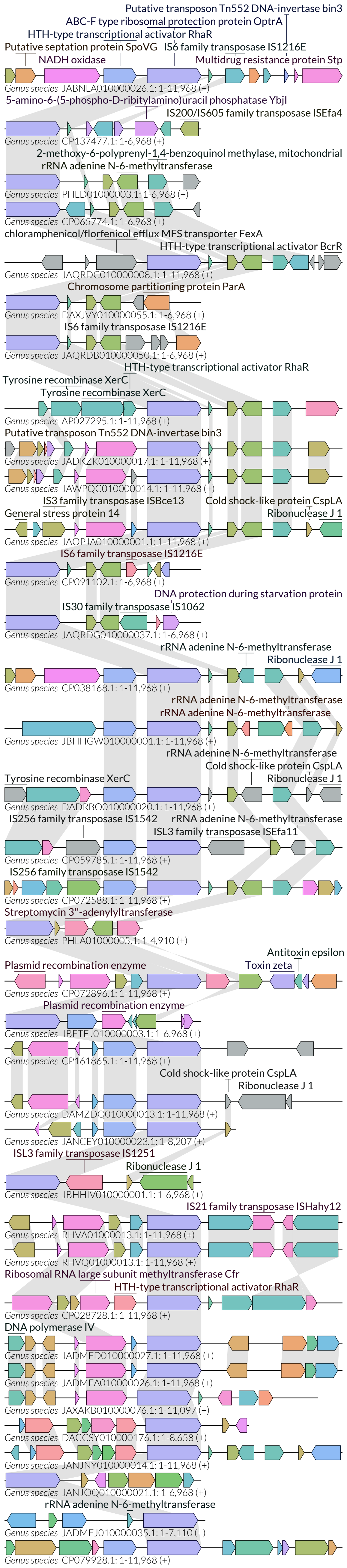
Putative transposon Tn552 DNA-invertase bin3
ABC-F type ribosomal protection protein OptrA
HTH-type transcriptional activator RhaR
Putative septation protein SpoVG
IS6 family transposase IS1216E
NADH oxidase
Multidrug resistance protein Stp
Genus species JABNLA010000026.1: 1-11,968 (+)
5-amino-6-(5-phospho-D-ribitylamino)uracil phosphatase YbjI
IS200/IS605 family transposase ISEfa4
Genus species CP137477.1: 1-6,968 (+)
2-methoxy-6-polyprenyl-1,4-benzoquinol methylase, mitochondrial
rRNA adenine N-6-methyltransferase
Genus species PHLD01000003.1: 1-6,968 (+)
Genus species CP065774.1: 1-6,968 (+)
chloramphenicol/florfenicol efflux MFS transporter FexA
HTH-type transcriptional activator BcrR
Genus species JAQRDC010000008.1: 1-11,968 (+)
Chromosome partitioning protein ParA
Genus species DAXJVY010000055.1: 1-6,968 (+)
IS6 family transposase IS1216E
Genus species JAQRDB010000050.1: 1-6,968 (+)
HTH-type transcriptional activator RhaR
Tyrosine recombinase XerC
Tyrosine recombinase XerC
Genus species AP027295.1: 1-11,968 (+)
Putative transposon Tn552 DNA-invertase bin3
Genus species JADKZK010000017.1: 1-11,968 (+)
Genus species JAWPQC010000014.1: 1-11,968 (+)
IS3 family transposase ISBce13
Cold shock-like protein CspLA
General stress protein 14
Ribonuclease J 1
Genus species JAOPJA010000001.1: 1-11,968 (+)
IS6 family transposase IS1216E
Genus species CP091102.1: 1-6,968 (+)
DNA protection during starvation protein
IS30 family transposase IS1062
Genus species JAQRDG010000037.1: 1-6,968 (+)
rRNA adenine N-6-methyltransferase
Ribonuclease J 1
Genus species CP038168.1: 1-11,968 (+)
rRNA adenine N-6-methyltransferase
rRNA adenine N-6-methyltransferase
Genus species JBHHGW010000001.1: 1-11,968 (+)
rRNA adenine N-6-methyltransferase
Cold shock-like protein CspLA
Tyrosine recombinase XerC
Ribonuclease J 1
Genus species DADRBO010000020.1: 1-11,968 (+)
IS256 family transposase IS1542
rRNA adenine N-6-methyltransferase
ISL3 family transposase ISEfa11
Genus species CP059785.1: 1-11,968 (+)
IS256 family transposase IS1542
Genus species CP072588.1: 1-11,968 (+)
Streptomycin 3''-adenylyltransferase
Genus species PHLA01000005.1: 1-4,910 (+)
Antitoxin epsilon
Plasmid recombination enzyme
Toxin zeta
Genus species CP072896.1: 1-11,968 (+)
Plasmid recombination enzyme
Genus species JBFTEJ010000003.1: 1-6,968 (+)
Genus species CP161865.1: 1-11,968 (+)
Cold shock-like protein CspLA
Ribonuclease J 1
Genus species DAMZDQ010000013.1: 1-11,968 (+)
Genus species JANCEY010000023.1: 1-8,207 (+)
ISL3 family transposase IS1251
Ribonuclease J 1
Genus species JBHHIV010000001.1: 1-6,968 (+)
IS21 family transposase ISHahy12
Genus species RHVA01000013.1: 1-11,968 (+)
Genus species RHVQ01000013.1: 1-11,968 (+)
Ribosomal RNA large subunit methyltransferase Cfr
HTH-type transcriptional activator RhaR
Genus species CP028728.1: 1-11,968 (+)
DNA polymerase IV
Genus species JADMFD010000027.1: 1-11,968 (+)
Genus species JADMFA010000026.1: 1-11,968 (+)
Genus species JAXAKB010000076.1: 1-11,097 (+)
Genus species DACCSY010000176.1: 1-8,658 (+)
Genus species JANJNY010000014.1: 1-11,968 (+)
Genus species JANJOQ010000021.1: 1-6,968 (+)
rRNA adenine N-6-methyltransferase
Genus species JADMEJ010000035.1: 1-7,110 (+)
Genus species CP079928.1: 1-11,968 (+)
