# Supplementary material for: Global distribution and genomic characteristics of linezolid resistance gene-positive Enterococcus faecium among humans, animals, and the environment
Source: Front Microbiol. 2026 Aug 10;17:1916332. doi: 10.3389/fmicb.2026.1916332 (PMC13501226; doi:10.3389/fmicb.2026.1916332)
Supplement: Supplementary file 2 [file Image_2.PDF]

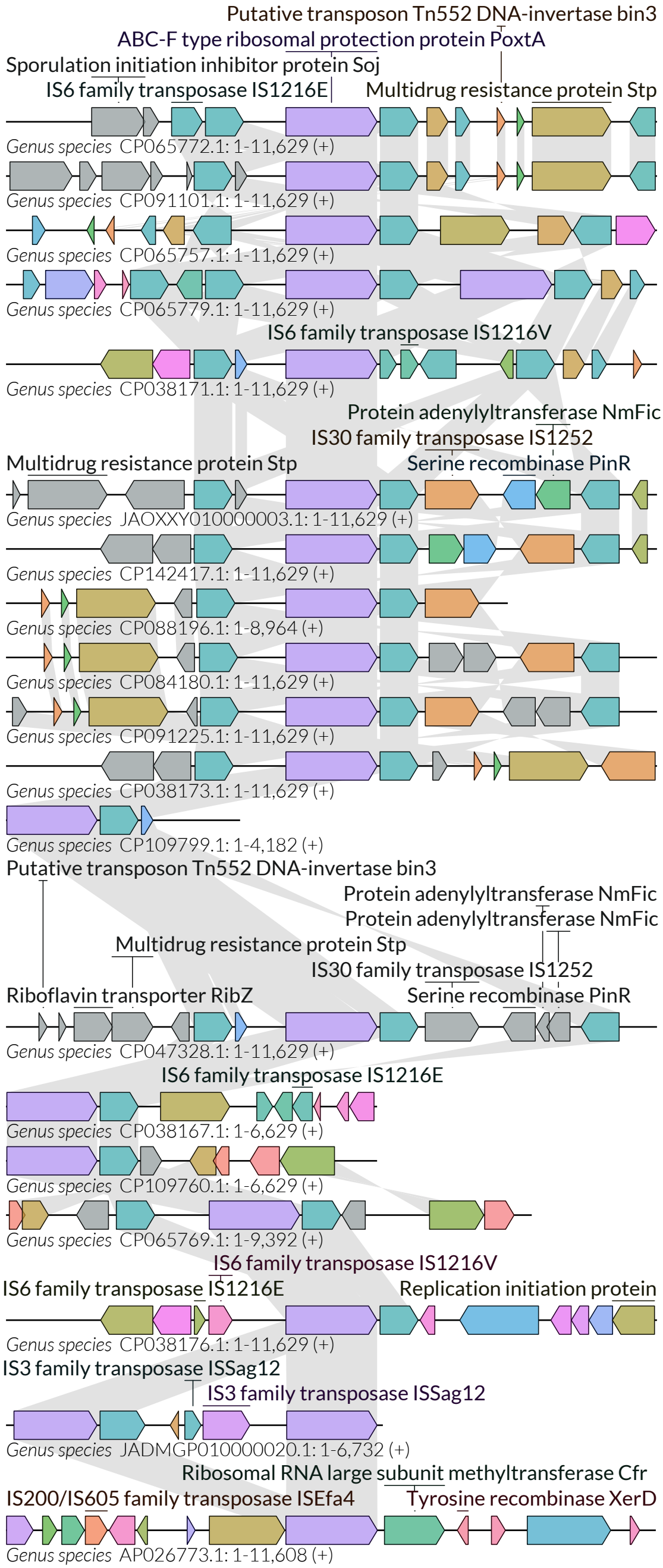
Putative transposon Tn552 DNA-invertase bin3
ABC-F type ribosomal protection protein PoxtA
Sporulation initiation inhibitor protein Soj
IS6 family transposase IS1216E
Multidrug resistance protein Stp
Genus species CP065772.1: 1-11,629 (+)
Genus species CP091101.1: 1-11,629 (+)
Genus species CP065757.1: 1-11,629 (+)
Genus species CP065779.1: 1-11,629 (+)
IS6 family transposase IS1216V
Genus species CP038171.1: 1-11,629 (+)
Protein adenylyltransferase NmFic
IS30 family transposase IS1252
Multidrug resistance protein Stp
Serine recombinase PinR
Genus species JAOXXY010000003.1: 1-11,629 (+)
Genus species CP142417.1: 1-11,629 (+)
Genus species CP088196.1: 1-8,964 (+)
Genus species CP084180.1: 1-11,629 (+)
Genus species CP091225.1: 1-11,629 (+)
Genus species CP038173.1: 1-11,629 (+)
Genus species CP109799.1: 1-4,182 (+)
Putative transposon Tn552 DNA-invertase bin3
Protein adenylyltransferase NmFic
Protein adenylyltransferase NmFic
Multidrug resistance protein Stp
IS30 family transposase IS1252
Riboflavin transporter RibZ
Serine recombinase PinR
Genus species CP047328.1: 1-11,629 (+)
IS6 family transposase IS1216E
Genus species CP038167.1: 1-6,629 (+)
Genus species CP109760.1: 1-6,629 (+)
Genus species CP065769.1: 1-9,392 (+)
IS6 family transposase IS1216V
IS6 family transposase IS1216E
Replication initiation protein
Genus species CP038176.1: 1-11,629 (+)
IS3 family transposase ISSag12
IS3 family transposase ISSag12
Genus species JADMGP010000020.1: 1-6,732 (+)
Ribosomal RNA large subunit methyltransferase Cfr
IS200/IS605 family transposase ISEfa4
Tyrosine recombinase XerD
Genus species AP026773.1: 1-11,608 (+)
