# Supplementary material for: Global distribution and genomic characteristics of linezolid resistance gene-positive Enterococcus faecium among humans, animals, and the environment
Source: Front Microbiol. 2026 Aug 10;17:1916332. doi: 10.3389/fmicb.2026.1916332 (PMC13501226; doi:10.3389/fmicb.2026.1916332)
Supplement: Supplementary file 3 [file Image_3.PDF]

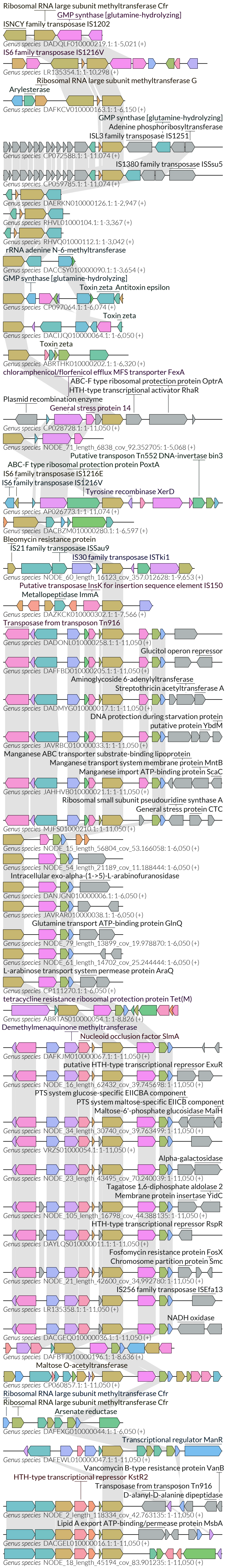
Ribosomal RNA large subunit methyltransferase Cfr
GMP synthase [glutamine-hydrolyzing]
ISNCY family transposase IS1202
Genus species DADQLF010000219.1: 1-5,021 (+)
IS6 family transposase IS1216V
Genus species LR135354.1: 1-10,298 (+)
Ribosomal RNA large subunit methyltransferase G
Arylesterase
Genus species DAFKCV010000163.1: 1-6,150 (+)
GMP synthase [glutamine-hydrolyzing]
Adenine phosphoribosyltransferase
ISL3 family transposase IS1251
Genus species CP072588.1: 1-11,074 (+)
IS1380 family transposase ISSsu5
Genus species CP059785.1: 1-11,074 (+)
Genus species DAERKN010000126.1: 1-2,947 (+)
Genus species RHVL01000104.1: 1-3,367 (+)
Genus species RHVQ01000112.1: 1-3,042 (+)
rRNA adenine N-6-methyltransferase
Genus species DACCSY010000090.1: 1-3,654 (+)
GMP synthase [glutamine-hydrolyzing]
Toxin zeta
Antitoxin epsilon
Genus species CP097064.1: 1-6,074 (+)
Toxin zeta
Genus species DACIJQ010000064.1: 1-6,050 (+)
Toxin zeta
Genus species ABRTHK010000202.1: 1-6,320 (+)
chloramphenicol/florfenicol efflux MFS transporter FexA
ABC-F type ribosomal protection protein OptrA
HTH-type transcriptional activator RhaR
Plasmid recombination enzyme
General stress protein 14
Genus species CP028728.1: 1-11,050 (+)
Genus species NODE_71_length_6838_cov_92.352705: 1-5,068 (+)
Putative transposon Tn552 DNA-invertase bin3
ABC-F type ribosomal protection protein PoxtA
IS6 family transposase IS1216E
IS6 family transposase IS1216V
Tyrosine recombinase XerD
Genus species AP026773.1: 1-11,074 (+)
Genus species DACBZM010000280.1: 1-6,597 (+)
Bleomycin resistance protein
IS21 family transposase ISSau9
IS30 family transposase ISTki1
Genus species NODE_60_length_16123_cov_357.012628: 1-9,653 (+)
Putative transposase InsK for insertion sequence element IS150
Metallopeptidase ImmA
Genus species DAZKCK010000302.1: 1-7,566 (+)
Transposase from transposon Tn916
Genus species DADONL010000258.1: 1-11,050 (+)
Glucitol operon repressor
Genus species DAFFBD010000205.1: 1-11,050 (+)
Aminoglycoside 6-adenylyltransferase
Streptothricin acetyltransferase A
Genus species DADMYG010000017.1: 1-11,050 (+)
DNA protection during starvation protein
putative protein YbdM
Genus species JAVRBC010000033.1: 1-11,050 (+)
Manganese ABC transporter substrate-binding lipoprotein
Manganese transport system membrane protein MntB
Manganese import ATP-binding protein ScaC
Genus species JAHHVB010000021.1: 1-11,050 (+)
Ribosomal small subunit pseudouridine synthase A
General stress protein CTC
Genus species MJFS01000210.1: 1-11,050 (+)
Genus species NODE_15_length_56804_cov_53.166058: 1-6,050 (+)
Genus species NODE_54_length_21189_cov_11.188444: 1-6,050 (+)
Intracellular exo-alpha-(1->5)-L-arabinofuranosidase
Genus species DANJGN010000006.1: 1-6,050 (+)
Genus species JAVRAR010000038.1: 1-6,050 (+)
Glutamine transport ATP-binding protein GlnQ
Genus species NODE_79_length_13899_cov_19.978870: 1-6,050 (+)
Genus species NODE_61_length_14702_cov_25.244444: 1-6,050 (+)
L-arabinose transport system permease protein AraQ
Genus species CP111270.1: 1-6,050 (+)
tetracycline resistance ribosomal protection protein Tet(M)
Genus species ABRTAS010000054.1: 1-8,826 (+)
Demethylmenaquinone methyltransferase
Nucleoid occlusion factor SlmA
Genus species DAFKJM010000067.1: 1-11,050 (+)
putative HTH-type transcriptional repressor ExuR
Genus species NODE_16_length_62432_cov_39.745698: 1-11,050 (+)
PTS system glucose-specific EIICBA component
PTS system maltose-specific EIICB component
Maltose-6'-phosphate glucosidase MalH
Genus species NODE_34_length_30740_cov_39.763499: 1-11,050 (+)
Genus species VRZS01000054.1: 1-11,050 (+)
Alpha-galactosidase
Genus species NODE_23_length_43495_cov_70.240039: 1-11,050 (+)
Tagatose 1,6-diphosphate aldolase 2
Membrane protein insertase YidC
Genus species NODE_105_length_16798_cov_44.388135: 1-11,050 (+)
HTH-type transcriptional repressor RspR
Genus species DAYLQS010000011.1: 1-11,050 (+)
Fosfomycin resistance protein FosX
Chromosome partition protein Smc
Genus species NODE_21_length_42600_cov_34.992780: 1-11,050 (+)
IS256 family transposase ISEfa13
Genus species LR135358.1: 1-11,050 (+)
NADH oxidase
Genus species DACGEQ010000036.1: 1-11,050 (+)
Genus species DAFBTJ010000196.1: 1-8,636 (+)
Maltose O-acetyltransferase
Genus species CP060857.1: 1-11,050 (+)
Ribosomal RNA large subunit methyltransferase Cfr
Ribosomal RNA large subunit methyltransferase Cfr
Arsenate reductase
Genus species DAFEXG010000044.1: 1-6,050 (+)
Transcriptional regulator ManR
Genus species DAFEWL010000047.1: 1-11,050 (+)
Vancomycin B-type resistance protein VanB
HTH-type transcriptional repressor KstR2
Transposase from transposon Tn916
D-alanyl-D-alanine dipeptidase
Genus species NODE_2_length_118334_cov_42.763135: 1-11,050 (+)
Lipid A export ATP-binding/permease protein MsbA
Genus species DACGEL010000016.1: 1-11,050 (+)
Genus species NODE_18_length_45194_cov_83.901235: 1-11,050 (+)
